# Effect of Deformation Mode on Grain Characteristics and Strength–Toughness of Al-Zn-Mg-Cu Alloy

**DOI:** 10.3390/ma17184633

**Published:** 2024-09-21

**Authors:** Guohui Shi, Mingyang Yu, Kai Zhu, Weicai Ren, Kai Wen, Xiwu Li

**Affiliations:** 1 State Key Laboratory of Nonferrous Metals and Processes, China GRINM Group Co., Ltd., Beijing 100088, China; 2 General Research Institute for Nonferrous Metals, Beijing 100088, China; 3 GRIMAT Engineering Institute Co., Ltd., Beijing 101407, China; 4 Northeast Light Alloy Co., Ltd., Harbin 150060, China

**Keywords:** rolling method, grain characteristics, fracture toughness

## Abstract

The Al-Zn-Mg-Cu alloy plate is a structural material widely used in aerospace, and its rolling process plays a crucial role in determining its performance. This study investigated the effects of different pass combinations of forward and spread rolling on the grain characteristics, strength, and fracture toughness of Al-Zn-Mg-Cu aluminum alloy plates under industrial conditions. The results show that initially using a small pass reduction followed by a larger one can improve the grain width and thickness on the Long Transverse–Short Transverse surface. Additionally, increasing the spread rolling pass enhances the grain width-to-thickness ratio on the TS surface. Performance tests indicate that grain characteristics have minimal influence on room-temperature tensile properties. However, a higher grain width-to-thickness ratio significantly improves the alloy’s fracture toughness.

## 1. Introduction

Al-Zn-Mg-Cu alloys are distinguished by their high specific strength, cost-effectiveness, corrosion resistance, and fatigue resistance, thereby establishing them as the predominant aluminum alloys in aviation [1,2,3,4]. Commonly used aluminum alloy structural parts for aircraft include plates, forgings, profiles, etc., of which plates are the most widely used aluminum alloy products. As a result, the processing and manufacturing techniques of aluminum alloy plates have consistently drawn significant interest from scholars globally.

Thermal rolling deformation serves as the primary processing technique for aluminum alloy plates, significantly impacting both the microstructure and properties of the alloy [5,6,7,8]. Sun et al. [9] conducted a study on the influence of thermal deformation temperature (ranging from 330 °C to 480 °C) during multi-pass hot rolling on the microstructure and dislocation density of the 7A04 aluminum alloy. The results demonstrated that when the deformation temperature reached 430 °C, extensive recrystallization occurred within the alloy, leading to the lowest dislocation density and a significant reduction in coarse precipitates. Zarei et al. [10] explored the effect of rolling temperature, ranging from 250 °C to 350 °C, on the mechanical properties of the 7075 aluminum alloy. They found that the room temperature strength and ductility of the 7075 aluminum alloy decreased with an increase in rolling temperature. However, Rajabi et al. [11] discovered an opposite pattern in the 6061 alloy; as the temperature rose from 250 °C to 450 °C, the alloy’s strength and ductility gradually increased while the yield strength gradually decreased. They suggested that this was attributed to an increase in the area fraction of recrystallization in the alloy during high-temperature rolling, with particles rich in iron or copper undergoing dynamic precipitation. Yang et al. [12] investigated the effect of the deformation amount on the microstructure of the 7075 alloy. When the deformation amount increased to 40%, dynamic recrystallization facilitated grain refinement. Under large deformation amounts, coarse second particles were broken down and refined into chains, partially spheroidizing and dissolving into the matrix. The dislocation density after solution treatment decreases when the amount of deformation is less than 80%, while it increases when the amount of deformation reaches 80%. Furthermore, Kazemi-Navaee [13] and Wang [14] et al. found that as the deformation amount increased, equiaxed grains became elongated along the rolling direction, gradually increasing the hardness and strength of the alloy but reducing its plasticity. In addition to studying the overall effect of deformation amount, Ju [15] also investigated the impact of pass reduction amount on the properties of 7075 plates. He found that the mechanical properties of the RD direction of the three passes (5%, 11% and 16%) were similar. Moreover, the larger the pass reduction, the better the mechanical properties in the TD direction.

While the impact of rolling temperature and deformation amount has been thoroughly researched, it is important to note that the hot rolling process of plates requires multiple passes to complete. Therefore, the total number of rolling passes and the deformation amount per pass significantly affect the microstructure characteristics. Despite Mondal et al. [16] having discovered that different hot cross-rolling methods can alter the internal texture of the alloy, relevant research remains scarce. There is a lack of studies on how the total number of hot rolling passes and single-pass reduction amount affect the grain characteristics of the Al-Zn-Mg-Cu alloy, which is crucial for regulating the strength and fracture toughness of the alloy. In summary, this paper aims to explore under industrial conditions how rolling parameters, including rolling direction, pass reduction amount, and total number of rolling passes, impact the grain characteristics and strength–toughness of the Al-Zn-Mg-Cu alloy. This study holds great practical significance for finely regulating microstructure and optimizing comprehensive properties during the industrial production process of the Al-Zn-Mg-Cu alloy.

## 2. Materials and Methods

**Figure 1:**
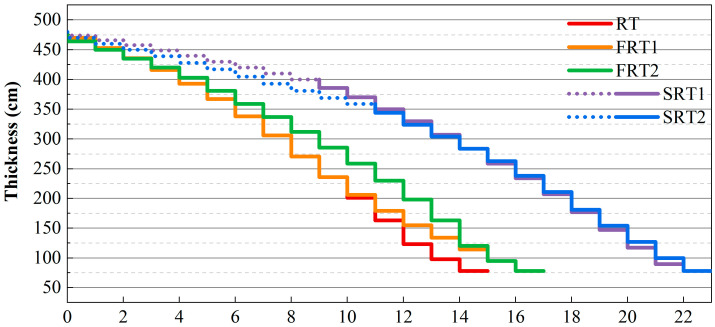
Process diagram of the plates.

The materials used in this study were Al-Zn-Mg-Cu alloy ingots prepared under industrialized conditions, with their chemical composition shown in Table 1, which were tested by Inductively Coupled Plasma-Atomic Emission Spectrometry. Ingots of the same specifications were subjected to forward rolling with different rolling pass distribution schemes and reverse rolling with varying width expansions to ultimately obtain thick plates of 78mm. Subsequently, the thick plates underwent identical solution and aging treatments to avoid differences arising from heat treatment. Figure 1 presents the rolling scheme for the thick plates, where RT is the basic process, FRT1 and FRT2 are the forward rolling technique (FRT), and SRT1 and SRT2 are the spread rolling technique (SRT). Moreover, 15 passes are used for the RT process, with large deformations applied in passes 12~15. The FRT1 process consists of 17 passes. Compared to the RT process, the 1st to 10th passes are the same, and from the 11th pass onwards, the single-pass deformation of the FRT1 process decreases, and the number of passes increases. The FRT2 process also consists of 17 passes, with smaller per-pass deformations in passes 2~12 and larger per-pass deformations in passes 13~17 compared to the FRT1 process. The SRT1 process is a 23-passes rolling process, in which the ingot is rolled to a thickness of 400 mm along the width direction in passes 1~9, followed by unidirectional rolling from the 10th pass, resulting in a final plate width of 2.2 m. The SRT2 process also involves 23 passes, with the ingot rolled to a thickness of 359 mm along the width direction in passes 1~11, followed by unidirectional rolling from the 12th pass, leading to a final plate width of 2.5 m.

The grain structures of the alloy under different rolling processes were characterized using EBSD (JSM-7900F, EDAX Inc., Pleasanton, CA, USA). The samples were mechanical polished and then electrolytically polished in a solution of 10% perchloric acid and 90% anhydrous ethanol at 30 V and room temperature. After that, the quantitative statistics of the grain characteristics were carried out using Image Pro Plus (IPP) software (version 6.0). For each process, 5 regions were counted, and from this, we obtained an average. The room temperature tensile properties of the materials were carried out on a microcomputer servo electronic universal testing machine at a stretching rate of 2 mm/min. Fracture toughness tests were conducted on a hydraulic servo fatigue machine using C[T] specimens with a thickness of 20 mm according to ASTM E399. The room temperature tensile properties and fracture toughness tests were carried out using 3 and 2 specimens, respectively, and average values were taken.

## 3. Results and Discussion

**Figure 2:**
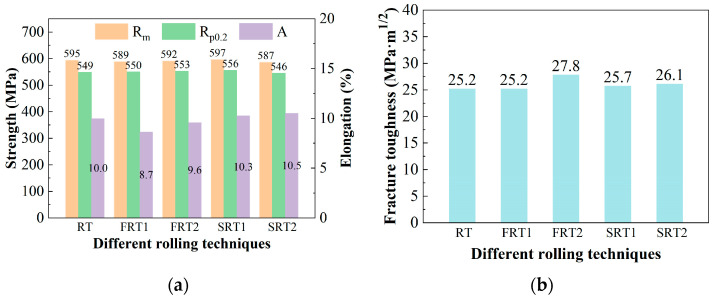
The properties of forward rolling plate: (**a**) tensile properties of L direction; (**b**) fracture toughness of L-T direction.

Figure 2 shows the room-temperature tensile properties in the L-direction and the fracture toughness results in the L-T direction of the plate. A comparison of the room-temperature tensile properties in the L direction reveals that the difference in the ultimate tensile strength and the yield strength of the alloys for the five processes is less than 10 MPa, while the difference in elongation is about 1%, showing a minor difference. This indicates that changes in the deformation process of the alloy have no significant impact on the room-temperature tensile properties in the L-direction of the alloy. Observing Figure 2b, it is found that the fracture toughness of the FRT1 process alloy is the same as that of the RT process alloy, both being 25.2 MPa·m^1/2^, while the fracture toughness of the FRT2 alloy significantly increased to 27.8 MPa·m^1/2^, which is an improvement of 10.32%. As for the spread rolling process, the fracture toughness of the alloys from the RT process to the SRT2 process increased by no more than 0.5 MPa·m^1/2^, indicating that the two spread rolling processes have a smaller impact fracture toughness of the alloys in the L-T direction.

**Figure 3:**
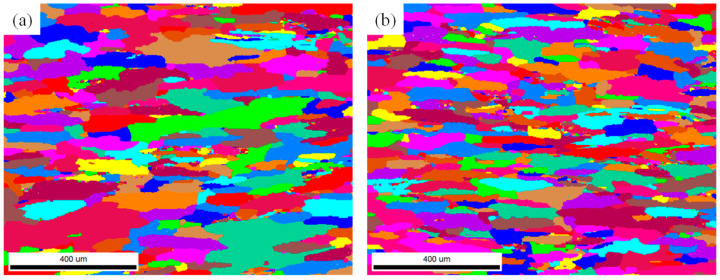

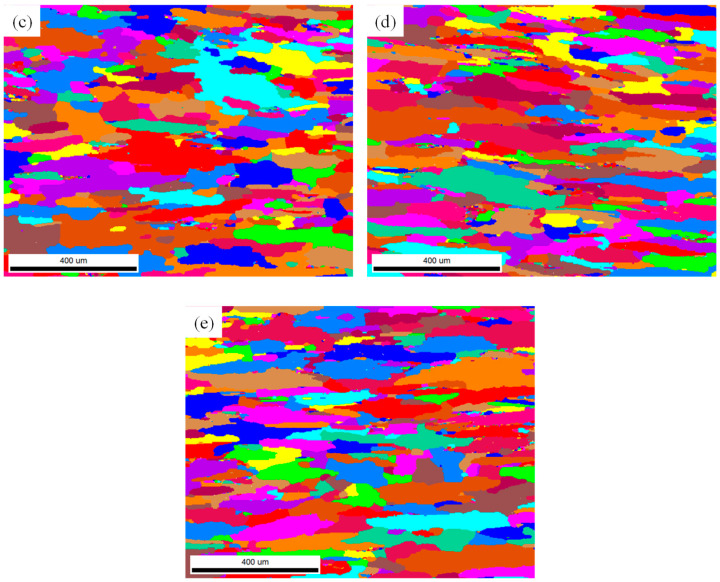
The EBSD pictures on TS surface at position D/2 of the plate: (**a**) RT; (**b**) FRT1; (**c**) FRT2; (**d**) SRT1; (**e**) SRT2.

Figure 3 shows the EBSD images of the Long Transverse–Short Transverse (TS) surface at the D/2 position (i.e., the cross-section at the center of the thickness) of the plates processed by different deformation techniques. It could be observed that the grains of the five deformation process alloys are mainly in a fibrous morphology, elongated along the T direction, while some small-sized equiaxed grains are discretely distributed in the alloys. Comparing the grain characteristics of the alloys processed by forward rolling, it was found that the grain width and thickness of the alloy deformed by the FRT1 process are smaller than those of the alloys deformed by the RT and FRT2 processes, whereas the grain width and thickness of the alloys deformed by the RT and FRT2 processes show no significant difference. As for the alloys processed by spread rolling techniques, the grain size in the thickness direction of the alloy deformed by the basic RT process is slightly larger than that of the alloys deformed by the other two spread rolling processes, with a minor difference in the width direction. Meanwhile, the grain width and thickness between the alloys deformed by the SRT1 and SRT2 processes are basically the same.

**Figure 4:**
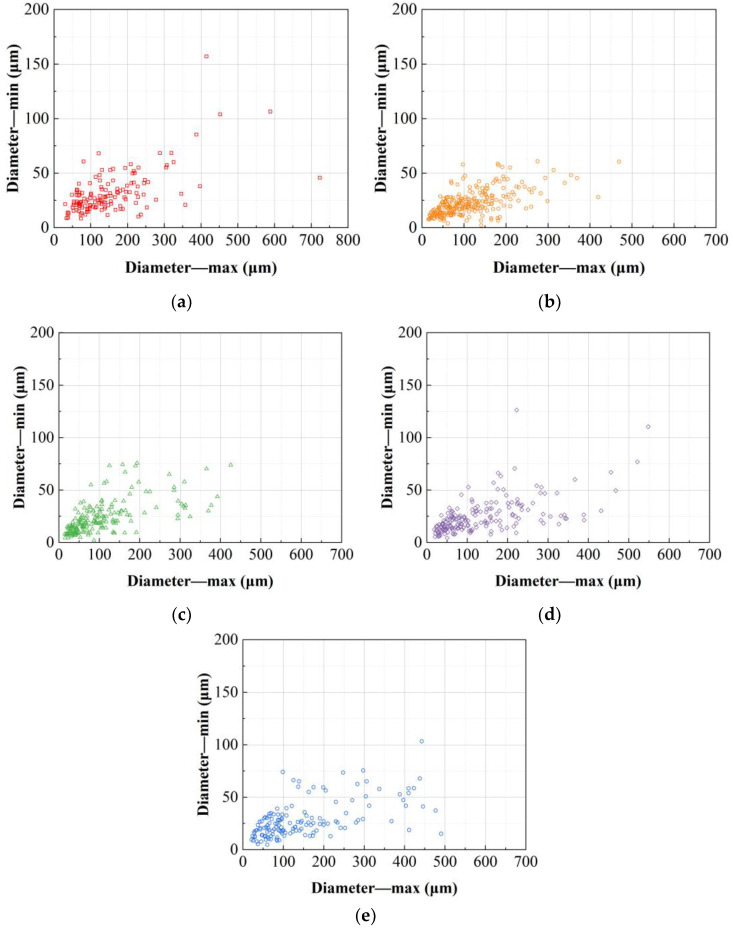
The grain size distribution on TS surface at position D/2 of the plate: (**a**) RT; (**b**) FRT1; (**c**) FRT2; (**d**) SRT1; (**e**) SRT2.

The grain width and thickness of the alloys under the five deformation processes were statistically analyzed as shown in Figure 4. As can be seen, the grain sizes of the alloys deformed by the five deformation processes are mainly distributed within the range of widths from 0 μm to 200 μm and thicknesses from 0 μm to 50 μm. Further analysis of the distribution of grain widths and thicknesses in each alloy reveals that the grain size distribution of the alloy deformed by the RT process is more dispersed, with some grains having widths greater than 500 μm or thicknesses greater than 100 μm. The overall grain size of the alloy deformed by the FRT1 process decreases, making the grain distribution more concentrated, with the thickest grain size being around 50 μm and the width not exceeding 500 μm. The grain size width distribution under the FRT2 process, which adjusted the pass deformation, is similar to that of the alloy deformed by the FRT1 process, but the thickness distribution range becomes larger, with many grains having thicknesses greater than 50 μm. Additionally, there is no significant difference in the thickness distribution of the alloy grains under the RT, SRT1, and SRT2 processes, while in terms of width, compared to the grains of the alloy deformed by the RT process, the grain distribution range of the SRT1 and SRT2 process alloys is larger, with the SRT1 process having some grains wider than 400 μm, and a greater number of grains with widths greater than 400 μm in the alloys deformed by the SRT2 process. This indicates that different spread rolling processes have little impact on the thickness of the alloy grains but significantly increase the width, with the SRT2 process alloy grains becoming more slender.

**Figure 5:**
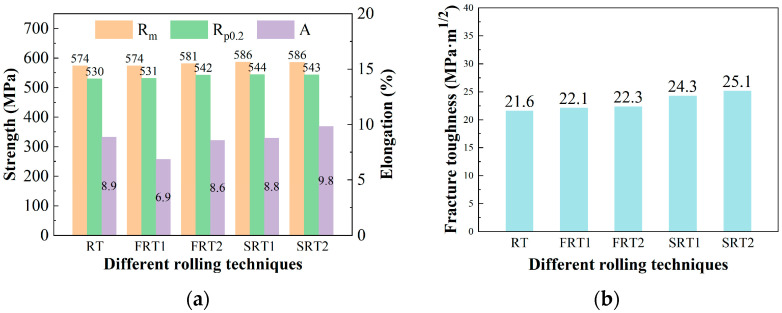
The properties of plate: (**a**) tensile properties of T direction; (**b**) fracture toughness of T-L direction.

Figure 5 shows the T-direction room temperature tensile properties and T-L-direction fracture toughness results of the plates. From Figure 5a, it can be seen that the ultimate tensile strength and the yield strength of the alloys deformed by the RT process and FRT1 process are essentially the same, but the elongation of the FRT1 process alloy is 2% lower than that of the RT process alloy. The ultimate tensile strength and the yield strength of the FRT2 process alloy are slightly higher than those of the RT and FRT1 process alloys, but the difference is small, and the elongation of the FRT2 process alloy is roughly equivalent to that of the RT process alloy. For the spread rolling processes SRT1 and SRT2, compared to the RT process, the differences in the ultimate tensile strength and the yield strength are both less than 15 MPa, and the variance in elongation does not exceed 1.0%, indicating a minor difference. This shows that changes in the deformation process of the alloy have no significant impact on the room-temperature tensile properties in the T-direction of the alloy. It is found from Figure 5b that the fracture toughness of the FRT1 process and FRT2 process alloys is essentially the same, slightly higher than that of the RT process alloy, but the difference is less than 1 MPa·m^1/2^, indicating that changes in the forward rolling deformation process have a smaller impact on the T-L direction fracture toughness of the alloy. However, from the RT process to the SRT1 process, the fracture toughness of the alloy increased by 2.7 MPa·m^1/2^, an improvement of 12.5%. In addition, the fracture toughness of the alloy increased by 0.8 MPa·m^1/2^ from the SRT1 process to the SRT2 process. This indicates that the spread rolling process significantly improves the fracture toughness of the alloy.

**Figure 6:**
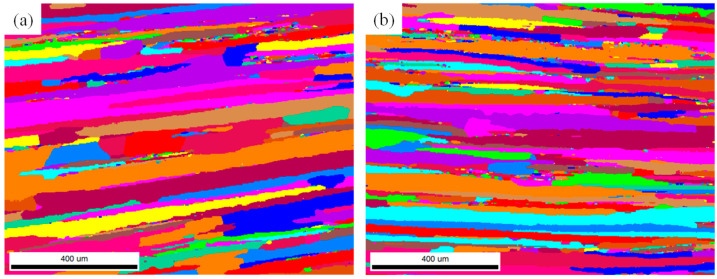

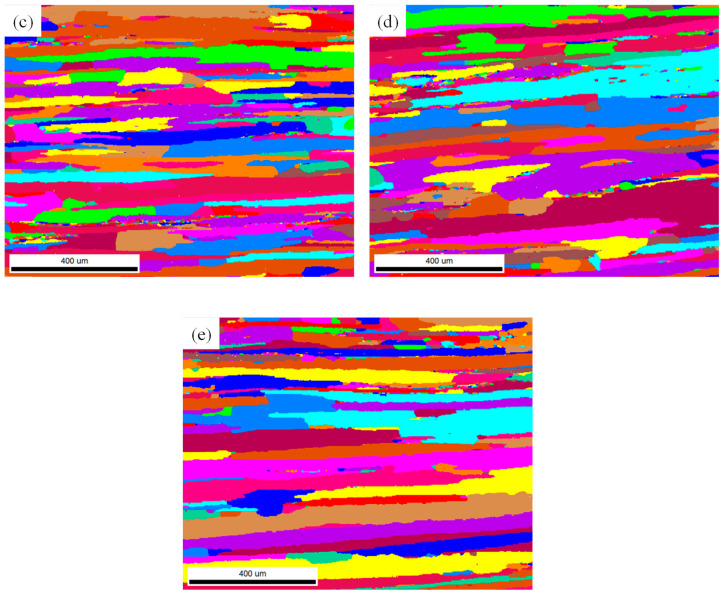
The EBSD pictures on LS surface at position D/2 of the plate: (**a**) RT; (**b**) FRT1; (**c**) FRT2; (**d**) SRT1; (**e**) SRT2.

Figure 6 shows the EBSD images of the LS plane at the D/2 position of the plates processed by different deformation techniques. Observation of the pictures shows that the grains of the five deformation process alloys are mainly in a fibrous morphology, elongated along the L direction, while there are some small-sized equiaxed grains distributed in the alloys in chains along the L direction. Comparing the size differences in the alloys of each process, it is found that the grain thicknesses of the alloys deformed by the FRT1 process and the FRT2 process are not significantly different, and are slightly smaller than that of the total alloy of the RT process. The grain thicknesses of the alloys deformed by the SRT2 process are smaller than that of the alloys deformed by the SRT1 process, which is basically the same as that of the alloys deformed by the RT process.

**Figure 7:**
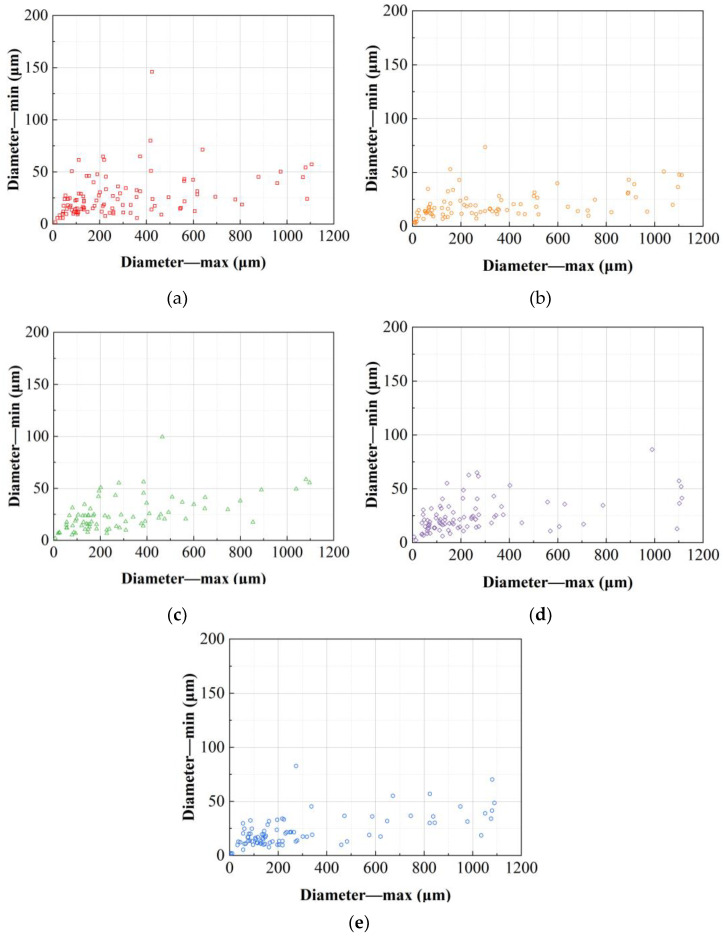
The grain size distribution on LS surface at position D/2 of the plate: (**a**) RT; (**b**) FRT1; (**c**) FRT2; (**d**) SRT1; (**e**) SRT2.

The width and thickness of the grains of the alloys under the five deformation processes were counted, and the results are shown in Figure 7. As can be seen from the figures, the grain size of the alloys deformed by the five deformation processes is mainly concentrated in the range of length 0 μm to 300 μm and thickness 0 μm to 50 μm, with the maximum grain length reaching approximately 1100 μm. Comparing the distribution of grain lengths and thicknesses among the alloys, it is found that the grain size distribution of the alloy deformed by the RT process is more dispersed, with a significantly higher number of grains thicker than 50 μm compared to the FRT1 and FRT2 process alloys. The grain thickness distribution of the alloys deformed by the FRT1 and FRT2 processes shows little difference, but the FRT2 process alloy has slightly fewer grains longer than 500 μm compared to the FRT1 process alloy. Compared to the RT process, the alloy deformed by the SRT1 process has fewer grains thicker than 50 μm, with little difference in width distribution. In contrast, there is little variance in the thickness distribution of grains between the alloys deformed by the SRT2 and RT process, but the SRT2 process alloys shows a slight decrease in the number of grains with grain lengths greater than 400 μm compared to the RT process alloys.

**Figure 8:**
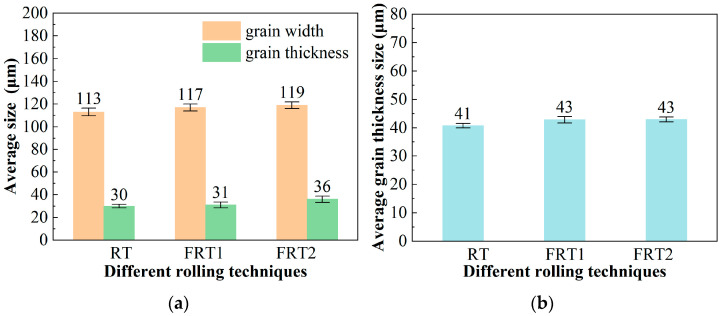
The grain characteristics at position D/2 of the plate: (**a**) average width/thickness on TS surface; (**b**) average grain thickness on LS surface.

The aforementioned study indicates that rolling processes have no significant impact on the alloy’s strength as the strength is primarily influenced by precipitates. Changes in the rolling process do not affect the characteristics of the precipitates, so the differences are not significant. In contrast, there are noticeable differences in fracture toughness. Figure 8 shows the statistical graphs of grain characteristics of the TS and LS surfaces of the alloy for the forward rolling process. Figure 8a presents the average grain width and thickness statistics of the three deformation processes alloys. Comparing the average width and thickness of grains among the three process alloys, it is found that the average width and thickness of the alloy grains under the RT process are slightly smaller than those of the FRT1 and FRT2 deformation process alloys. The average grain width and thickness of the FRT2 alloy are 2 μm and 5 μm larger than those of the FRT1 alloy, respectively. Figure 8b displays the statistical results of the average grain thickness for alloys under three deformation processes. It can be seen that the average grain thickness size of the FRT1 and FRT2 process alloys is the same, which is 43 μm, 2 μm larger than that of the RT process alloy. This indicates that there is no significant difference in grain size thickness under the three deformation processes. Combined with the performance test results, it is found that when the width/thickness characteristics of the alloy grains are essentially consistent, the grain size of the alloy increases, that is, when the number of thicker grains in the alloy increases, it is beneficial to improve the fracture toughness of the alloy.

**Figure 9:**
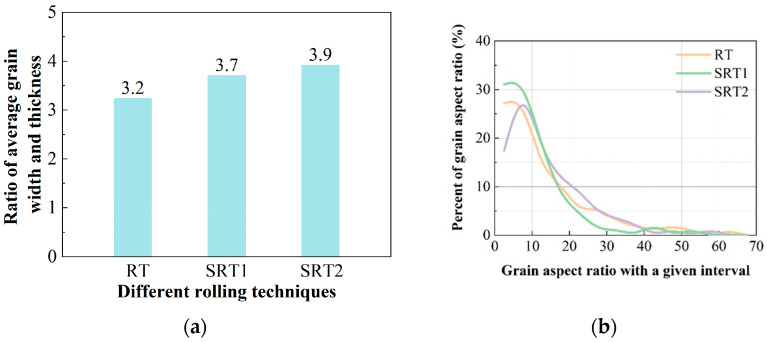
The grain characteristics at position D/2 of the plate: (**a**) average grain width/thickness ratio on TS surface; (**b**) distribution of grain aspect ratio on LS surface.

Figure 9a shows the statistical results of the average width/thickness ratio of grains on the TS surface of the three deformation processes. As can be seen from the figure, when the deformation process changes from RT to SRT1, the average width/thickness ratio of the alloy increases from 3.2 to 3.7, an increase of 0.5. From the SRT1 process to the SRT2 process, the average width/thickness ratio of the alloy increases by 0.2. This indicates that with a change in the deformation process, the grains gradually become more elongated and the fibrous characteristics become more pronounced. Figure 9b presents the statistical results of the width/thickness ratio distribution of grains on the LS surface for the three alloys. The grain aspect ratio distribution curves of the three alloys have the same trend, mainly distributed between 0 and 10, and there are some grain width/thickness ratios greater than 10. Comparing the peak positions of the grain aspect ratio distribution curves of the alloys under three deformation processes, it is found that compared to the RT process alloy, the curve peak of the SRT2 process alloy shifts to the right, broadening the range of width/thickness ratio distributions. This means that the proportion of grains with larger width/thickness ratios significantly increases and the grains become more elongated. From the SRT1 to SRT2 process, the curve peak of the SRT1 process alloy clearly shifts to the left, indicating that the proportion of grains with smaller width/thickness ratios increases, that is, the grain length decreases. This is because a further increase in width while the thickness remains unchanged, or more specifically, a decrease in the width/thickness ratio, leads to a decrease in length. Combined with the fracture toughness results, it is found that for spread rolling alloys, when the proportion of grains with large width/thickness ratios significantly increases, that is, the grains become slender, the fracture toughness of the corresponding alloy increases significantly. This is because that the fibrous grains possess better toughness than equiaxed grains and have a stronger ability to resist crack propagation [17,18].

## 4. Conclusions

In this paper, the effects of the rolling process on the grain structure, strength, and fracture toughness of the Al-Zn-Mg-Cu alloy were investigated by adjusting the forward and spread rolling processes with different pass deformations. The research demonstrates that improving the properties of the alloy by controlling its grain characteristics through the rolling process is both feasible and valuable. This provides guidance for the deformation of Al-Zn-Mg-Cu alloy plates. This study reaches the following conclusions:

(1) Compared to the basic RT process, the grain width and thickness on the TS surface of the FRT1 and FRT2 alloys, which underwent more rolling passes, increased, although the difference in the grains’ width-to-thickness ratio was not significant. The grain distribution characteristics on the LS surface were largely the same. The TS surface grains of the FRT2 alloy, which was initially rolled with a small pass reduction followed by a larger one, were slightly larger than those of the FRT1 alloy, which followed the opposite rolling sequence.
(2) Compared to the basic RT process, the width-to-thickness ratios of the grains gradually increased as the number of spread rolling passes increased from the SRT1 to the SRT2 process.
(3) The greater the grain width-to-thickness ratio, indicating more pronounced fibrous grain characteristics, the higher the alloy’s fracture toughness.

## Figures and Tables

**Table 1:** The chemical composition of the alloy (wt%).

Element	Zn	Mg	Cu	Zr	Fe	Si	Al
Content	6.40	2.38	2.35	0.12	0.08	0.06	Bal.

## Data Availability

The data are not publicly available due to privacy restrictions.
